# Co-enrollment of critically ill patients into multiple studies: patterns, predictors and consequences

**DOI:** 10.1186/cc11917

**Published:** 2013-01-08

**Authors:** Deborah Cook, Ellen McDonald, Orla Smith, Nicole Zytaruk, Diane Heels-Ansdell, Irene Watpool, Tracy McArdle, Andrea Matte, France Clarke, Shirley Vallance, Simon Finfer, Pauline Galt, Tim Crozier, Rob Fowler, Yaseen Arabi, Clive Woolfe, Neil Orford, Richard Hall, Neill KJ Adhikari, Marie-Clauide Ferland, John Marshall, Maureen Meade

**Affiliations:** 1 Department of Medicine, McMaster University Health Sciences Center, Hamilton, ON L8N 3Z5, Canada; 2 Department of Clinical Epidemiology and Biostatistics, McMaster University Health Sciences Center, Hamilton, ON L8N 3Z5, Canada; 3 Interdepartmental Division of Critical Care, University of Toronto, Toronto, ON, Canada; 4 Department of Critical Care, Ottawa University, Ottawa, ON, Canada; 5 Intensive Care Unit, The Alfred, Melbourne, VIC, Australia; 6 The George Institute for Global Health, University of Sydney, Sydney, NSW, Australia; 7 Intensive Care Unit, Monash Medical Centre, Melbourne, VIC, Australia; 8 Intensive Care Department, King Saud Bin Abdulaziz University for Health Sciences, Riyadh, Saudi Arabia; 9 Intensive Care Unit, Royal Prince Alfred Hospital, Sydney, NSW, Australia; 10 Intensive Care Unit, Barwon Health, Geelong, VIC, Australia; 11 Departments of Anesthesiology, Medicine, Surgery and Pharmacology, Dalhousie University, Halifax, NS, Canada; 12 Intensive Care Unit, Hopital Laval, Quebec, QC, Canada

## Abstract

**Introduction:**

Research on co-enrollment practices and their impact are limited in the ICU setting. The objectives of this study were: 1) to describe patterns and predictors of co-enrollment of patients in a thromboprophylaxis trial, and 2) to examine the consequences of co-enrollment on clinical and trial outcomes.

**Methods:**

In an observational analysis of an international thromboprophylaxis trial in 67 ICUs, we examined the co-enrollment of critically ill medical-surgical patients into more than one study, and examined the clinical and trial outcomes among co-enrolled and non-co-enrolled patients.

**Results:**

Among 3,746 patients enrolled in PROTECT (Prophylaxis for ThromboEmbolism in Critical Care Trial), 713 (19.0%) were co-enrolled in at least one other study (53.6% in a randomized trial, 37.0% in an observational study and 9.4% in both). Six factors independently associated with co-enrollment (all *P *< 0.001) were illness severity (odds ratio (OR) 1.35, 95% confidence interval (CI) 1.19 to 1.53 for each 10-point Acute Physiology and Chronic Health Evaluation (APACHE) II score increase), substitute decision-makers providing consent, rather than patients (OR 3.31, 2.03 to 5.41), experience of persons inviting consent (OR 2.67, 1.74 to 4.11 for persons with > 10 years' experience compared to persons with none), center size (all ORs > 10 for ICUs with > 15 beds), affiliation with trials groups (OR 5.59, 3.49 to 8.95), and main trial rather than pilot phase (all ORs > 8 for recruitment year beyond the pilot). Co-enrollment did not influence clinical or trial outcomes or risk of adverse events.

**Conclusions:**

Co-enrollment was strongly associated with features of the patients, research personnel, setting and study. Co-enrollment had no impact on trial results, and appeared safe, acceptable and feasible. Transparent reporting, scholarly discourse, ethical analysis and further research are needed on the complex topic of co-enrollment during critical illness.

## Introduction

**Figure 1:**
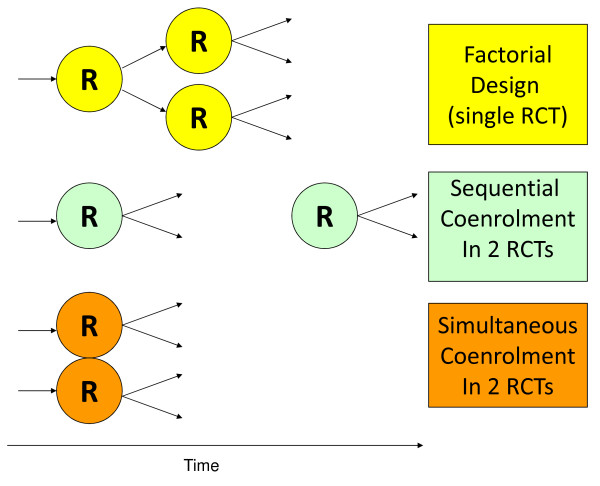
**Factorial and co-enrollment designs**. In this figure, we present a schematic for a factorial design randomized trial, sequential co-enrollment in two randomized trials and simultaneous co-enrollment in two randomized trials.

Clinical trials are essential to improve care and reduce morbidity and mortality in the intensive care unit (ICU). Some critically ill patients are eligible for more than one study. Restricting enrollment to only one study when patients are eligible for more than one is a potentially modifiable barrier to recruitment [1]. Testing two interventions concurrently can be achieved with a factorial design as used successfully by the Acute Respiratory Distress Syndrome Network. In other circumstances, when trials are initiated by different investigators at different times, with different inclusion and exclusion criteria, co-enrollment can facilitate either sequential or simultaneous recruitment (Figure 1).

Co-enrollment in multiple trials, often driven by patient demand, occurs in persons with human immunodeficiency virus (HIV) [2], and was documented among 23% of persons with HIV in six ongoing studies [3]. In this population, co-enrollment is actively encouraged by some research programs [3] but not others [2]. In pre-hospital resuscitation trials, co-enrollment occurs either in series or in parallel [4]. Half of the members of two critical care research consortia reported co-enrollment of a patient in more than one study in the last year [5]. In a parental survey, 74% endorsed enrollment of their premature babies in 2 or more studies, 50% would consent to 3 or more studies, and 10% were willing to join more than 10 studies [6].

Some Institutional Review Boards restrict the practice of co-enrollment, while concerned about patient safety, decisional burden or scientific integrity. Given the dearth of evidence on these issues, trialists have called for consideration of co-enrollment on a case-by-case basis, and reporting on its impact [7]. The primary objective of this study was to document the patterns and predictors of patient co-enrollment in an international heparin thromboprophylaxis trial. The secondary objective was to examine the consequences of co-enrollment on clinical and trial outcomes.

## Materials and methods

PROTECT (Prophylaxis for ThromboEmbolism in Critical Care Trial) (clinicaltrials.gov NCT00182143) was a randomized, blinded clinical trial comparing unfractionated heparin to dalteparin for thromboprophylaxis [8]. Patients considered eligible were ≥ 18 years old, weighed > 45 kilograms, and were expected to remain in ICU > 72 hours. Exclusion criteria were admission diagnosis of trauma, neurosurgery or orthopedic surgery, need for therapeutic anticoagulation, receipt of > 72 hours of heparin, contraindication to heparin, blood or pork products, pregnancy, life support limitation, and prior enrollment in this or a related trial. The primary outcome was proximal leg deep vein thrombosis (DVT). Other outcomes were pulmonary embolism, venous thromboembolism, bleeding, heparin-induced thrombocytopenia, duration of mechanical ventilation, ICU and hospital stay, and ICU and hospital mortality. PROTECT was conducted over four years from May 2006 to June 2010 in 67 ICUs in Canada, the United States, the United Kingdom, Australia, Brazil and Saudi Arabia, as published previously [9].

Ethical approval was obtained from each participating Institutional Research Board (listed at the end of the manuscript under PROTECT Collaborators). In-person informed consent was required prior to randomization. Deferred consent was not permitted. For substitute decision-makers not in hospital, initial telephone consent, followed by in-person consent when possible, was approved in 16 of the 67 (23.9%) centers.

**Figure 2:**
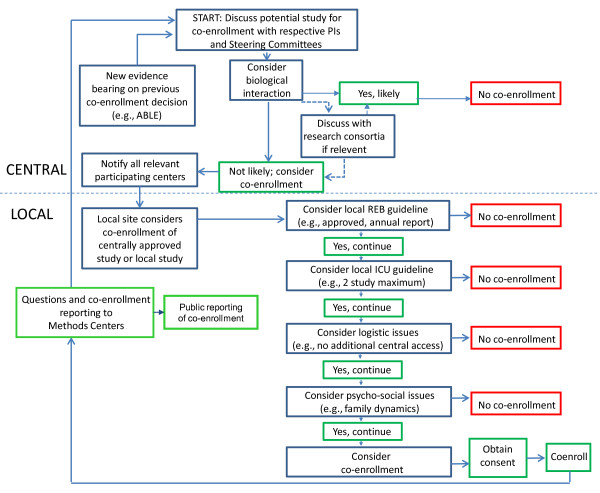
**Co-enrollment schema**. In this figure, we outline steps taken to consider co-enrollment of one patient into one or more additional studies. ABLE Trial, Age of Blood Evaluation Trial; ANZICS, Australian and New Zealand Intensive Care Society Clinical Trials Group; CCCTG, Canadian Critical Care Trials Group; Fonda, fondaparinux; REB, Research Ethics Board; UFH, unfractionated heparin.

Beginning and throughout the trial, the PROTECT Steering Committee reviewed each multicenter protocol to decide whether co-enrollment was admissible, using Canadian Critical Care Trials Group guidelines [10]. These guidelines outline important scientific (for example, interacting interventions), psychosocial (for example, family stress) and logistic (for example, research coordinator workload) factors to consider. The general approach to co-enrollment was that all reasonable efforts should be made to minimize the exclusion of patients co-enrolled in another trial if they would likely represent those patients to whom trial results would possibly be applied in practice, as long as biologic interaction of the interventions being tested in the two trials seemed highly implausible. Dialogue between the principal investigator and steering committees of each multicenter study determined whether co-enrollment would impact the scientific integrity of either study. When relevant, this was reasoned at the Canadian Critical Care Trials Group or the Australian and New Zealand Intensive Care Society Clinical Trials Group meetings for refutation or ratification. If co-enrollment was endorsed, each participating center handled the relevant study governed by formal or informal co-enrollment policies of their ICU or hospital Institutional Review Board. Local policies could deny co-enrollment approved centrally. Local, single-center study co-enrollment could also be approved after agreement with the PROTECT Steering Committee and the relevant consortium. Decisions were revisited if emerging evidence required reconsideration (Figure 2). All other studies into which patients were enrolled before, concurrent with, or subsequent to PROTECT were documented on case report forms.

One example was co-enrollment into the Age of Blood Evaluation Study (ABLE, ISRCTN44878718). ABLE is a randomized trial evaluating mortality following transfusion of red blood cells stored up to one week versus stored up to 42 days [11]. Both PROTECT and ABLE investigators initially endorsed co-enrollment. Months later, an observational trauma study suggested that among a subgroup of patients transfused with more than five units, when patients received blood stored less than, versus more than, 28 days, DVT rates (16.7% versus 34.5%, *P *= 0.006), and mortality rates (13.9% versus 26.7% *P *= 0.02) were lower [12]. If prolonged blood storage is thrombophilic in trauma, this could similarly increase DVT risk in critically ill medical-surgical patients. In reconsidering PROTECT and ABLE co-enrollment, we sought additional evidence.

Using an existing prospective observational study database of 261 medical-surgical ICU patients screened for DVT [13], we evaluated age of transfused blood as an additional DVT risk factor. We also examined red blood cell transfusion as a possible risk factor in this population because in 349 trauma patients, transfusions increased DVT risk [14]. We found that 126 (48.3%) patients had at least one transfusion, and patients had a median of four (interquartile range; IQR 2, 8) units. Multivariable analyses documented that neither red blood cell transfusion nor storage age predicted DVT in medical-surgical patients. Trends were counter to findings in trauma (for example, red blood cells stored for ≤ 7 days had a higher associated DVT risk compared to > 7 days (hazard ratio 5.3; 95% CI 1.3 TO 22.1)) [15]. Based on inconclusive research evidence, the PROTECT and ABLE Steering Committees affirmed co-enrollment into these trials. Given the PROTECT sample size, we anticipated similar transfusion rates and similar age of red blood cells transfused in the two arms. The ABLE trial now includes venous thromboembolism as a tertiary outcome.

### Statistical analysis

We reported proportions with 95% confidence intervals (CI), and mean and standard deviation (SD) or median and IQR. We compared groups using Chi square, t-test and Fisher's Exact test. We examined univariate associations between co-enrollment rates (the dependent variable) and other factors (independent variables) related to characteristics of the patient, research coordinator, center and trial. A *P-*value of < 0.01 was considered statistically significant.

We conducted multivariable logistic regression analyses. To avoid incorporation of highly correlated independent variables into the model, we selected one of four measures of research coordinator experience, one of three measures of research infrastructure, and research consortium affiliation rather than country. The following independent factors were analyzed: patient factors (age, sex, Acute Physiology and Chronic Health Evaluation (APACHE) II score, medical versus surgical status); individual consenting (substitute decision-maker or patient); research coordinator factors (years of experience obtaining consent for studies in the ICU when PROTECT began); center factors (number of ICU beds; number of full time research staff; national research consortium affiliation (Canadian Critical Care Trials Group or the Australian or New Zealand Intensive Care Society Clinical Trials Group); and year (pilot trial or year 1, 2, 3 and 4 of the full trial). Results are summarized using odds ratios (OR) with 95% CI. A *P-*value of < 0.01 was considered statistically significant.

We calculated the proportion of patients in each arm of the PROTECT trial who were co-enrolled. To evaluate whether co-enrollment affected patient safety, we re-analyzed the proportion of patients in each arm who had serious adverse events. To evaluate whether trial results would be any different without co-enrolled patients, we re-analyzed overall results excluding these patients.

## Results

In 67 participating ICUs, 3,746 patients were enrolled in PROTECT. Consent was declined for 810 patients. Patients who were not enrolled in PROTECT due to enrollment in another study represented 65 of 2,288 patients (2.8%) who were eligible but not randomized. Those 65 patients were enrolled in 71 other studies, 41 (63.1%) of which were industry-funded.

Among the 3,746 patients in PROTECT, 713 (19.0%) were co-enrolled in at least one other study (53.6% in a randomized trial, 37.0% in an observational study and 9.4% in both types of studies). Co-enrollment rates across participating centers ranged from 0 to 53.9% and across participating countries from 1.1 to 26.0%. No co-enrollment occurred in 30 of 67 (44.8%) centers.

Factors associated with co-enrollment in univariate analysis are presented in Table 1. Patients with higher illness severity and medical conditions were more likely to be co-enrolled than patients who were less ill and surgical. Substitute decision-makers were more likely to agree to co-enrollment than patients. Research coordinators with more ICU experience, and those with more experience obtaining consent in the ICU, were more likely to co-enroll patients than those with less experience. Centers with more ICU beds and centers affiliated with national research consortia were more likely to co-enroll than others. A higher proportion of patients were co-enrolled in Canada, the United States and Australia than in Brazil, Saudi Arabia and the United Kingdom. Co-enrollment was less common in the pilot phase of the trial than the main trial.

**Table 1:** Characteristics of factors associated and not associated with co-enrollment

	Total patients (*N *= 3,746)	Not co-enrolled (*N *= 3,033)	Co-enrolled patients (*N *= 713)	*P*-value
**Patient characteristics**				
**Age, mean (SD)**	61.4 (16.5)	61.4 (16.6)	61.4 (15.9)	**0.996**
Female, N (%)*	1,614 (43.3)	1,319 (43.8)	295 (41.4)	**0.257**
APACHE II score, mean (SD)**	21.5 (7.8)	21.1 (7.8)	23.3 (7.6)	**< 0.001**
**Medical admission type, N (%)**	2,831 (75.6)	2,262 (74.6)	569 (79.8)	**0.004**
Person consenting, N (%)***				
**Patient**	354 (9.5)	335 (11.1)	19 (2.7)	**< 0.001**
**Substitute decision-maker**	3,380 (90.5)	2,686 (88.9)	694 (97.3)	
**Research coordinator characteristics**				
**Years of non-research ICU experience, N (%)**				
**0 years**	572 (15.3)	491 (16.2)	81 (11.4)	**0.003**
**> 0 to 10 years**	1,254 (33.5)	1,017 (33.5)	237 (33.2)	
**> 10 years**	1,920 (51.3)	1,525 (50.3)	395 (55.4)	
**Years of procuring consent for clinical studies in ICU, N (%)**				
**0 years**	302 (8.1)	268 (8.8)	34 (4.8)	**< 0.001**
**> 0 to 10 years**	2,652 (70.8)	2,276 (75.0)	376 (52.7)	
**> 10 years**	792 (21.1)	489 (16.1)	303 (42.5)	
**Center characteristics**				
**Number of ICU beds screened, N (%)**				
**< 15 beds**	420 (11.2)	414 (13.6)	6 (0.8)	**< 0.001**
**15 to 20 beds**	1622 (43.3)	1,218 (40.2)	404 (56.7)	
**> 20 beds**	1,704 (45.5)	1,401 (46.2)	303 (42.5)	
**Full time ICU research staff, N (%)**				
**< 1 FTE**	286 (7.6)	280 (9.2)	6 (0.8)	**< 0.001**
**1 FTE**	740 (19.8)	622 (20.5)	118 (16.5)	
**> 1 FTE**	2,720 (72.6)	2,131 (70.3)	589 (82.6)	
**Formal trials group affiliation, N (%)**				
**Yes**	3,224 (86.1)	2,533 (83.5)	691 (96.9)	**< 0.001**
**No**	522 (13.9)	500 (16.5)	22 (3.1)	
**Country, N (%)**				
**Canada**	2,456 (65.6)	1,818 (59.9)	638 (89.5)	**< 0.001**
**Australia**	768 (20.5)	715 (23.6)	53 (7.4)	
**Brazil**	275 (7.3)	272 (9.0)	3 (0.4)	
**Saudi Arabia**	138 (3.7)	135 (4.5)	3 (0.4)	
**United States**	91 (2.4)	77 (2.5)	14 (2.0)	
**United Kingdom**	18 (0.5)	16 (0.5)	2 (0.3)	
**Characteristic of enrollment phase, N (%)**				
**Pilot trial**	128 (3.4)	126 (4.2)	2 (0.3)	**< 0.001**
**Year 1**	556 (14.8)	504 (16.6)	52 (7.3)	
**Year 2**	826 (22.1)	626 (20.6)	200 (28.1)	
**Year 3**	1,009 (26.9)	761 (25.1)	248 (34.8)	

**Year 4**	**1,227 (32.8)**	**1,016 (33.5)**	**211 (29.6)**	

In this table, we compare patients, person consenting, approaches to consent and research environment associated with co-enrollment versus no co-enrollment. Formal trials group affiliation refers to a center associated with either the Canadian Critical Care Trials Group or the Australian and New Zealand Intensive Care Society Clinical Trials Group. *P*-values refer to results of univariate analyses. APACHE II score, Acute Physiology and Chronic Health Evaluation II score; ICU, intensive care unit; SD, standard deviation.

In Table 2, we present the six factors independently associated with co-enrollment in the multivariable analysis. In order of decreasing strength of association, these were: phase of the trial (all ORs > 8 for recruitment beyond the pilot phase); center affiliation with a research consortium (OR 5.59, 3.49 to 8.95); center size (all ORs > 10 for ICUs with > 15 beds); substitute decision-makers providing consent rather than patients (OR 3.31, 2.03 to 5.41); experience of research coordinator (OR 2.67, 1.74 to 4.11 for > 10 years of experience compared to persons whose first trial was PROTECT); and patient illness severity (odds ratio (OR), 95%CI 1.35 (1.19 to 1.53 for each 10-point increase in APACHE II score).

**Table 2:** Factors independently associated with co-enrollment in multivariate analysis

	Odds ratio (95% CI)	*P-*value
**Patient demographics**		
**Age (10-year increase)**	0.96 (0.91, 1.02)	**0.155**
**Female**	0.92 (0.77, 1.11)	**0.403**
**APACHE II score (10-point increase)**	1.35 (1.19, 1.53)	**< 0.001**
**Medical versus surgical**	1.26 (1.01, 1.57)	**0.041**
**Individual consenting**		
**Substitute decision-maker versus patient**	3.31 (2.03, 5.41)	**< 0.001**
**Years of procuring consent for clinical studies in ICU**		
**> 0 to 10 years versus 0 years**	0.83 (0.55, 1.25)	**< 0.001**
**> 10 years versus 0 years**	2.67 (1.74, 4.11)	
**Center size (beds screened for PROTECT patients)**		
**15 to 20 beds versus < 15 beds**	20.06 (7.56, 53.25)	**< 0.001**
**> 20 beds versus < 15 beds**	13.76 (5.15, 36.80)	
**Full time ICU research staff**		
**1 FTE versus < 1 FTE**	1.13 (0.41, 3.11)	**0.966**
**> 1 versus < 1 FTE**	1.10 (0.40, 3.03)	
**Formal trials group affiliation**		
**Yes versus no**	5.59 (3.49, 8.95)	**< 0.001**
**Year of PROTECT**		
**Year 1 versus Pilot**	8.22 (1.95, 34.61)	**< 0.001**
**Year 2 versus Pilot**	32.89 (7.95, 135.98)	
**Year 3 versus Pilot**	38.15 (9.24, 157.51)	

**Year 4 versus Pilot**	**24.53 (5.94, 101.25)**	

In this table, we present the independent factors associated with co-enrollment of one patient into two or more studies identified by multivariate regression analysis, presented using odds ratios (OR) and 95% confidence intervals (95% CI). *P*-values refer to results of multivariate analyses. ANZICS, Australian and New Zealand Intensive Care Society Clinical Trials Group; APACHE II score, Acute Physiology and Chronic Health Evaluation II score; CCCTG, Canadian Critical Care Trials Group; FTE, full time equivalent; ICU, intensive care unit.

Table 3 summarizes characteristics of the studies into which PROTECT patients were co-enrolled. The majority were co-enrolled into another academic investigator-initiated study (97.5%). Of 713 patients, 592 (83.0%) were co-enrolled in one other study, 93 (13.0%) were co-enrolled in two studies, and 28 (3.9%) were co-enrolled in three or more studies.

**Table 3:** Co-enrollment study characteristics.

	N (% of 713 patients)
**Type of other study**	
**Randomized trial**	**380 (53.3)**
**Observational study**	**265 (37.2)**
**Both**	**68 (9.5)**
**Genesis of other study**	
**Investigator-initiated**	**695 (97.5)**
**Industry-initiated**	**15 (2.1)**
**Both**	**3 (0.4)**
**CCCTG or ANZICS affiliated study**	
**Yes**	**451 (63.3)**
**No**	**181 (25.4)**
**Both**	**81 (11.4)**
**Number of studies into which patients were co-enrolled**	
**1**	**591 (82.9)**
**2**	**94(13.2)**
**3**	**25 (3.5)**

4*	**3 (0.4)**

In this table, among the 713 patients who were co-enrolled in another study, we present the co-enrollment study type (randomized trial, observational study), study genesis (investigator-initiated versus industry-initiated), affiliation with a research consortia, and the number of studies into which PROTECT patients were co-enrolled. The bottom half of the table outlines, of the 865 co-enrollments, which studies were involved. ANZICS, Australian and New Zealand Intensive Care Society Clinical Trials Group; CCCTG, Canadian Critical Care Trials Group.

Of 865 co-enrollments involving 713 patients, the most common other international trials tested pharmaconutrition, intensive glucose control, sedation interruption and high frequency oscillation (Table 1). Observational studies were both quantitative (for example, registries, audits, quality improvement studies, diagnostics, translational biology or long-term follow-up studies), and qualitative (for example, interviews, focus groups).

The proportion of patients co-enrolled in any study was similar between the dalteparin group and the unfractionated heparin group (352 (18.8%) versus 361 (19.3%), *P *= 0.74). There were no differences between groups in patients enrolled in any randomized trial (209 (11.2%) versus 239 (12.8%), *P *= 0.14), or the proportion in each group enrolled in any of the five most common co-enrollment studies (197 (10.5%) versus 223 (11.9%), *P *= 0.20). Twenty PROTECT patients were co-enrolled in ABLE (9 of 1,873 (0.5%) in the dalteparin group and 11 of 1,873 (0.6%) in the unfractionated heparin group), *P *= 0.82.

Among patients co-enrolled in other randomized trials, rates of serious adverse events were similar between the dalteparin (2 of 209, 1.0%) and unfractionated heparin (0 of 239, 0.0%) groups, *P *= 0.14, as per the main trial findings (7 of 1,873, 0.4%) versus 6 of 1,873, 0.3%), respectively, *P *= 0.74. Protocol violations were also similar (data not shown). In Table 4, we show that the overall PROTECT results excluding patients co-enrolled in other randomized trials, which were no different than the results of all patients randomized [9]. That is, pulmonary embolism rates were lower in patients receiving dalteparin compared to those receiving unfractionated heparin; rates of DVT, venous thrombosis and major bleeding were similar. No patients were withdrawn or lost to follow-up whether co-enrolled or not.

**Table 4:** PROTECT results excluding patients co-enrolled in another randomized trial

N (%)	Dalteparin (*N *= 1,873) All patients	UFH (*N *= 1,873) All patients	Hazard ratio (95%CI)	Dalteparin (*N *= 1,664) Not co-enrolled in RCT	UFH (*N *= 1,633) Not co-enrolled in RCT	Hazard ratio (95%CI)
**Primary outcome: proximal leg deep vein thrombosis**	94 (5.1)	108 (5.9)	0.91 (0.68, 1.23)	83 (5.0)	93 (5.7)	**0.87 (0.63, 1.21)**
**Any pulmonary embolism**	22 (1.2)	42 (2.3)	0.48 (0.27, 0.84)	19 (1.1)	35 (2.1)	**0.48 (0.26, 0.89)**
**Any venous thromboembolism**	150 (8.2)	184 (10.0)	0.87 (0.69, 1.10)	133 (8.0)	161 (9.9)	**0.83 (0.64, 1.07)**
**Major bleeding**	100 (5.5)	105 (5.7)	0.98 (0.73, 1.31)	88 (5.3)	93 (5.7)	**0.94 (0.69, 1.28)**
**Heparin-induced thrombocytopenia**	5 (0.3)	12 (0.7)	0.47 (0.16, 1.37)	5 (0.3)	10 (0.6)	**0.56 (0.18, 1.67)**

**Hospital mortality**	**395 (21.7)**	**444 (24.3)**	**0.91 (0.79, 1.05)**	**372 (22.4)**	**391 (23.9)**	**0.95 (0.82, 1.10)**

In this table we report the main results of the original trial including all patients, and results including only those patients, (209 in the dalteparin group and 239 in the unfractionated heparin group), who were not co-enrolled in another randomized trial (1,664 in the dalteparin arm and 1,633 in the unfractionated heparin arm). CI, confidence interval; RCT, randomized clinical trial; UFH, unfractionated heparin.

## Discussion

In this international heparin thromboprophylaxis trial, one-fifth of patients were co-enrolled in at least one other study. Half of the co-enrollments were in randomized trials, although a variety of study designs were involved. Co-enrollment was limited to one or two additional studies in 83% and 13% of patients, respectively. These findings are consistent with membership surveys of research consortia indicating that two was the median number of randomized trials into which one patient was enrolled [5].

Multivariate analysis showed that consent encounters with substitute decision-makers were more likely to involve co-enrollment than those with patients. This suggests that more seriously ill patients are frequently eligible for several studies, yet too sick to make decisions themselves, congruent with the finding that patients who were co-enrolled were more seriously ill than those who were not. Substitute decision-makers may seek several research opportunities while helping to advance science, so-called conditional altruism [16].

Research coordinators with greater consent experience were more likely to co-enroll than others, suggesting that professional maturity may foster sound judgment about approaching persons for co-enrollment, whether training enhances comfort and success with co-enrollment is unclear. Co-enrollment occurred more often in larger ICUs, and in centers affiliated with a national consortium, perhaps reflecting group norms. More frequent during the full trial than the pilot phase, facility with co-enrollment may have increased over time.

Participation in another study was the reason why 65 eligible patients were not enrolled in PROTECT; 63% of these studies were industry-initiated. Only 2% of PROTECT patients were co-enrolled in industry studies. Generally, industry-funded trials, compared to other trials, are more likely to exclude individuals due to age, comorbidities and concomitant medications, raising concerns about their generalizability [17]. However, if industry trials prohibit co-enrollment in academic studies, selection bias in academic trials may result, as well as slower completion, thereby delaying answers to publicly motivated research questions. Certainly, co-enrollment in trials of investigational drugs or devices is imprudent due to difficulty monitoring safety and interpreting harm. Since patients in the investigator-informed, industry-funded trial comparing drotrecogin alfa to placebo in patients with persistent septic shock (PROWESS-SHOCK, NCT00604214) [18] would typically receive heparin thromboprophylaxis in the absence of contraindications, PROTECT co-enrollment was permitted. We identified three patients who were eligible for PROTECT but not recruited because of enrollment in PROWESS-SHOCK, and no PROTECT patients who were co-enrolled in PROWESS-SHOCK.

One major focus regarding permissible co-enrollment in two academic trials is the biologic plausibility of the two interventions having a potentiating or attenuating effect on each other. Having identical primary outcomes in two academic trials would not be a sole criterion for prohibiting co-enrollment, especially when treatment effects are expected to be modest, which is common in critical care. For example, if two trials had the same primary outcome of mortality (for example, a trial comparing starch resuscitation vs normal saline in septic shock and intensive insulin therapy vs liberal glucose management in heterogenous ICU patients), the two interventions would likely be considered unrelated, and co-enrollment would be permitted, because starch and antioxidants would not be known to mediate their effect on mortality through related mechanisms. Several additional scientific issues need careful consideration regarding co-enrollment, such as projected impact on statistical power, outcome ascertainment bias, increased risk of adverse events and the ultimate interpretation of study results. We recommend widespread consultation about the merits and demerits of various co-enrollment pairs before and during conduct of a trial.

Furthermore, specific approaches to data collection and analysis can be established *a priori *if concerns exist about co-enrollment. Shared definitions and case report forms for use across studies could help [3], as we used for PROTECT and ABLE. New research influencing previous co-enrollment decisions should prompt revisiting previous decisions as studies unfold. Documenting consecutive and concurrent co-enrollment eligibility and consent rates throughout a trial, and transparent reporting of co-enrollment upon completion, including effect modification and risk of harm, will help to disseminate co-enrollment patterns, and provide data to examine its actual rather than perceived impact. If concerns exist and trialists are willing to exchange randomization codes, unadjusted and adjusted analyses can be conducted to evaluate the impact not just of substantial co-enrollment of patients in one trial on the results of the other trial, and vice versa, but also the impact of each specific allocation arm.

Although an understanding of all available treatment options is important for informed consent for medical therapy, there is no similar perception that patients should be informed of all available studies for which they are eligible. Indeed, most human subjects research discourse deals with protection from harm rather than opportunity for participation [1], which is particularly germane to co-enrollment. More open discussion will help to elucidate key ethical issues, since the vulnerability of critically ill patients [19] raises concern about adverse effects from co-enrollment. We found that PROTECT patients co-enrolled in randomized trials were not more likely to have serious adverse events or protocol violations compared to patients who were not co-enrolled. *Post hoc *analyses of PROTECT omitting patients co-enrolled in randomized trials yielded the same overall results as the main analysis. Finally, no PROTECT patients were lost to follow-up, thus, no co-enrolled patients were withdrawn.

In 2007, a tri-national survey showed that only 11% of respondents indicated that their local Institutional Review Board had a co-enrollment policy, whereas 35% reported a local ICU guideline [5]. Co-enrollment guidelines exist for adult resuscitation studies [4], pediatric [20] and adult [10] critical care. Public health mandates to answer research questions quickly during pandemics have encouraged co-enrollment of patients in treatment and observational studies [21]. Professional position statements about whether, when, why and how to co-enroll will raise awareness and facilitate stakeholder dialogue.

Limitations of this study include our inability to explore the decisional burden on substitute decision-makers, patients and research coordinators. However, in another four-month single center study, we found consent rates similar for any single enrollment (84%) and co-enrollment (79%) opportunity [22]. We could not document rates or reasons for no co-enrollment, or which person declined (for example, patient, substitute decision-maker, physician, surgeon, anesthesiologist). Examining the choice of which study to pursue if a patient was eligible for more than one was beyond the scope of this project. However, investigators report that when approaching persons for co-enrollment in a randomized trial, they consider trial rigor and relevance, potential for benefit or harm, consortium affiliation and remuneration [5].

Strengths of this study include comprehensive documentation of co-enrollment throughout a multicenter trial. Investigators used a prospective, transparent framework for co-enrollment decisions, independently examining each pair of studies, guided by independent Institutional Review Boards, and research consortia. Using multivariate analysis, significant predictors of co-enrollment were identified, adjusting for confounding. We examined the impact on patient and trial outcomes. Representation from diverse ICUs and countries enhances the generalizability of these findings, which may apply to other academic trials testing currently available interventions.

## Conclusions

Co-enrollment was common in this thromboprophylaxis trial, and was strongly associated with specific features of the patients, research personnel, setting and study. Co-enrollment was an effective, feasible method to enhance recruitment, provided that the patients or substitute decision-maker, clinicians, principal investigators, steering committees, research consortium and local Institutional Review Boards agreed. Co-enrollment did not influence overall trial results, patient safety or adverse events. Further scientific debate, ethical analysis and research are needed on the complex topic of co-enrollment for critically ill patients.

## Key messages

• In this international heparin thromboprophylaxis trial of 3,746 patients, one-fifth of patients were co-enrolled in at least one other study. Half of the co-enrollments were in randomized trials, although a variety of study designs were involved and almost all co-enrollments were in academic investigator-initiated studies.

• In decreasing strength of association, six factors were independently associated with co-enrollment: later phase of the trial compared to the pilot phase, center affiliation with a research consortium, larger center size, substitute decision-makers providing consent rather than patients, greater research coordinator experience and higher patient illness severity.

• Co-enrollment did not influence overall trial results, patient safety or adverse events.

• Before and during a trial, we suggest widespread consultation among investigators, clinicians, trial steering committees, research consortia and local Institutional Review Boards about the scientific, psychosocial and logistic effects of various co-enrollment pairs.

• Transparent reporting, scholarly discourse, ethical analysis and further research are needed on the complex topic of co-enrollment during critical illness.

## Abbreviations

ABLE: Age of Blood Evaluation; APACHE: Acute Physiology and Chronic Health Evaluation; CI: confidence interval; DVT: deep vein thrombosis; HIV: human immunodeficiency virus; ICU: intensive care unit; IQR: interquartile range; OR: odds ratio; PROTECT: Prophylaxis for ThromboEmbolism in Critical Care Trial; SD: standard deviation

## Competing interests

The authors declare that they have no competing interests.

## Authors' contributions

DC, EM, FC and NZ conceived of the study. DC, EM, FC, NZ, IW, TM, AM, FC and MCF designed the study. NZ and SV coordinated the study. DC obtained funding. EM, OS, NZ, IW, TM, AM, FC, SV and MCF collected data. DC, RF, NA, JM and MM consulted on methods. DHA performed the analyses. YA, NA, RF and DC designed the figure. DHA, DC, SF, TC, RF, YA, CW, NO, RH, NA, JM and MM interpreted the data. DC, EM, OS, DHA and MM wrote the draft. All authors read and approved the final manuscript for publication.
